# Seed-assisted synthesis of TS-1 crystals containing Al with high catalytic performances in cyclohexanone ammoximation†

**DOI:** 10.1039/c8ra10104c

**Published:** 2019-01-18

**Authors:** Yan Xue, Guangling Zuo, Yiqiang Wen, Huijuan Wei, Meng Liu, Xiangyu Wang, Baojun Li

**Affiliations:** Biological and Chemical Engineering College, Nanyang Institute of Technology 80 Changjiang Road Nanyang 473004 China yanxue800@163.com; Institute of Industrial Catalysis, School of Chemistry and Molecular Engineering, Zhengzhou University 100 Science Road Zhengzhou 450001 China xiangyuwang@zzu.edu.cn

## Abstract

In this study, titanium silicalite (TS-1) crystals containing Al were synthesized using aluminosilicate MFI zeolites as seeds in a tetrapropylammonium bromide (TPAB)–ethanolamine (EA) system. The TS-1 containing Al possessed large size, large *L*_*b*_ value and higher catalytic activity in cyclohexanone ammoximation. Larger *L*_*b*_ value would endow the TS-1 crystals with better mechanical strength and erosion resistance. The introduction of an Al atom into the TS-1 crystals resulted in the production of more acid sites and a bit strong Brönsted acid sites; these acid sites were more favorable to the catalytic performances in cyclohexanone ammoximation.

## Abstract 2

Using aluminosilicate zeolites seeds, Al atoms are introduced into the TS-1 crystals, resulting in high *L*_*b*_value and more acid sites, which improve the catalytic activity in cyclohexanone ammoximation.

## Graphical abstract

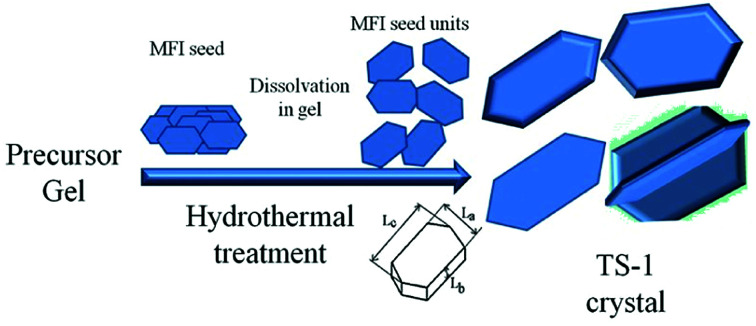

## 1. Introduction

TS-1 molecular sieves with an MFI topology structure have received significant attention due to their active and selective catalytic performances in many selective oxidation reactions^1^ such as hydroxylation of aromatics,^2,3^ epoxidation of alkenes,^4,5^ ammoximation of ketone,^6,7^ and oxidation of bulky molecules.^8^ For TS-1 crystals, due to their diffusion limitation, the catalytic properties are dependent on the size of pores along the *b*-axis.^9^ The deactivation behavior of the TS-1 catalyst is mainly caused by the deposits of coke on the micropores of zeolite.^10,11^ Therefore, many approaches were explored to solve the problem. The first effective strategy was to enlarge the size of the *b*-axis micropores; then, hierarchical TS-1 materials were synthesized using the mesopore template,^12–14^ and hierarchical porous structures were constructed by treating the TS-1 zeolite with organic bases.^15–17^ The second measure was to decrease the crystal size or reduce the *b*-axis depth; then, the diffusion limitations would be alleviated or even eliminated. The seed-assisted syntheses of zeolites could induce rapid nucleation, shorten crystallization time and decrease the zeolite crystal size.^18–22^ Furthermore, the b-oriented TS-1 zeolite membranes were synthesized by seed induction.^23–25^ However, in our previous study, after a 420 h continuous reaction for cyclohexanone ammoximation, many defects and caves were found on the surface of the big TS-1 particle, and some amorphous particles appeared on the outside of the TS-1 particles.^26^ The dissolution of Si in the basic reaction medium led to a decrease in the catalytic stability.^27^ It is still our pursuit to prepare the rather thick TS-1 with high catalytic performances and higher stability.

Bifunctional zeolite catalysts have been employed in various processes in the petroleum refining and petrochemical industries because of their metallic and acid sites.^28^ Some metals, such as precious metals,^29–31^ Cd^2+^,^32^ and Fe^2+^,^33^ incorporated into the TS-1 skeletons have improved the catalytic activity of TS-1. It has been reported that TS-1 with Al^3+^ showed excellent activity and selectivity in the epoxidation of alkenes.^34,35^

The liquid-phase ammoximation of ketone over titanium zeolites as a highly efficient technology is attracting attention from industrial sectors.^36,37^ Cyclohexanone oxime is a key intermediate for the production of ε-caprolactam as a starting feed for the manufacture of nylon-6. The hierarchical porous structure was much more effective in improving the catalytic properties of TS-1 zeolite for ketone ammoximation^38,39^ because the orifices on the surface of the catalysts could alleviate the diffusion limitations. Zhuo *et al.*^40^ demonstrated the relation between the Lewis acid strength in titanosilicate catalyst and the selectivity of oximes; TS-1 with stronger Lewis acidity provided better selectivity to oximes.

**Scheme 1:**
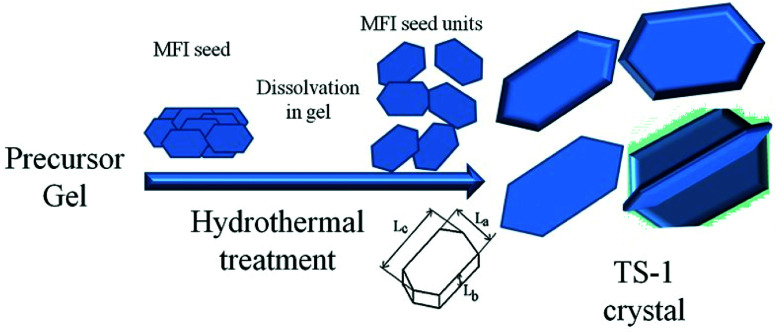
The synthesis process of TS-1 by MFI seeds.

TS-1 with large size and high *L*_*b*_ value would possess better mechanical strength and erosion resistance. In this study, large-sized TS-1 crystals were prepared from an inorganic reactant system using different sized seeds to control the size (Scheme 1). Aluminosilicate and pure silicon zeolites were used as seeds to prepare the TS-1 crystals and TS-1 containing Al. The resources of the Ti and Si template used in this study were cost-effective and suitable for industrialization production. The obtained zeolite particles were post-treated by alkaline solution and TPAB template to introduce mesopores into the zeolites; this eliminated the diffusion limitations of bulky molecules. The TS-1 using nano ZSM-5 seed exhibited big size, excellent catalytic activity and stability in cyclohexanone ammoximation.

## 2. Experimental

### 2.1 Preparation of seeds

Nano S-1 seed was synthesized with TEOS as the Si source. The molar composition of the precursors was set to 1.0TEOS : 0.20TPAOH : 20H_2_O. The alcohol contents of the mixture were removed by heat treatment at 70 °C for 2 h. The sol was aged at room temperature for 3 h. Subsequently, the resulting sol was subjected to hydrothermal treatment at 170 °C for 48 h. The solid product was filtered by centrifugation, washed with distilled water, and then dried at 60 °C for 12 h followed by calcination at 500 °C for 6 h. The micro S-1 seed was synthesized by the traditional hydrothermal treatment, and NaOH was used as a mineralizer. The molar composition of the precursors was 1.0SiO_2_ : 0.05TPAB : 0.20NaOH : 20H_2_O.

The seeds of ZSM-5:Al(OH)_3_ and silica aerogel are the Al and Si sources, and the molar composition of the mixture was 11.5Na_2_O : 100SiO_2_ : 3Al_2_O_3_ : 2750H_2_O. The nanosized TS-1 was added as seed. The solution was crystallized at 453 K for 48 h. After washing, filtration and drying, nano ZSM-5 seeds were obtained. In addition, the micro ZSM-5 was obtained when no seed was added in the abovementioned solution.

### 2.2 Synthesis

TS-1 was prepared by hydrothermal synthesis using colloidal silica (31 wt%) as the silicon source, tetrabutyl titanate (TBOT) as the titanium source and TPAB as the template. Ethanolamine (EA) was used to adjust the pH value of the matrix gel. Isopropyl alcohol (IPA) was used as an additive. Molar composition of the gel was SiO_2_ : 0.02TiO_2_ : 0.5IPA : 0.1TPAB : 20H_2_O. The process was similar to that reported in our early work.^26^ TS-1 was post-treated according to the report.^26^ The powders were modified with EA and TPAB, and 1.0 g of TS-1 raw powder was dispersed in EA and TPAB solution (molar composition of the solution was 1EA : 0.2TPAB : 50H_2_O, 12 mL). Moreover, two types of zeolites of pure silicon and aluminosilicate with the MFI structure were selected as seeds: nano S-1 (n-S-1) and micro S-1 (m-S-1) and nano ZSM-5 (n-ZSM-5) and micro ZSM-5 (m-ZSM-5). The corresponding TS-1 prepared using the four seeds of n-S-1, m-S-1, n-ZSM-5 and m-ZSM-5 were denoted as TS-1-*n* (*n* = 1–4) according to the abovementioned order. The solid product yields of TS-1 were 83.2% (TS-1-1), 85.3% (TS-1-2), 85.8% (TS-1-3), and 87.2% (TS-1-4).

### 2.3 Characterization of TS-1

X-ray diffraction (XRD) patterns were obtained using an automatic powder diffractometer (Xpert PRO, PANalytical B. V.) in transmission geometry with Cu Kα1 (*λ* = 1.5406 Å). The relative crystallinity degree (RCD) was calculated by comparing the total intensity of five characteristic peaks of each sample with the intensity of TS-1-1 as 100%.^39^ The preparation and treatment conditions before measurement were uniform. The data were obtained for the same amount of sample using the same sample holder under the same instrumental conditions. Fourier transform infrared (FTIR) spectra were obtained using a Thermo Nicolet IR 380 Spectrometer in the range from 2000 to 400 cm^−1^. UV-vis spectra were obtained using an Agilent Cary 5000 spectrometer from 200 to 410 nm. Nitrogen sorption measurements were performed at liquid nitrogen temperatures (77 K) using a Quantachrome NOVA 1000e surface area and pore size analyzer (Quantachrome Instrument, USA). Total specific surface area (*S*_BET_) and pore volume were calculated according to the multi-point Brunauer–Emmett–Teller (BET) based on the adsorption branch of isotherm curves and the *t*-plot method. Pore size calculations were performed with the NLDFT method based on the desorption branch of the isotherm curves.^41^ Scanning electron microscopy (SEM) images were obtained with the uncoated powders immobilized on the sample holders with gold paste using the field emission microscope FEI Quanta 250 FEG at 20.00 kV. The particle size distribution was measured using the laser particle size analyzer RISE-2008. Ammonia temperature-programmed desorption (TPD) was carried out using a self-assembled fixed-bed reactor connected to a thermal conductivity detector (TCD). The samples were first outgassed by thermal treatment from ambient temperature to 600 °C at the heating rate of 10 °C min^−1^ in an Ar stream (1.0 mL s^−1^). After cooling at 150 °C, the sample was saturated with a NH_3_ stream and consequently treated with an Ar stream (1.0 mL s^−1^) for 90 min. Finally, the temperature was increased to 560 °C at the heating rate of 10 °C min^−1^, while determining the NH_3_ desorption. The FTIR spectra of pyridine adsorbed to the zeolite samples were acquired using a Thermo Nicolet IR 380 Spectrometer. The samples were prepared as thin self-supporting wafers of 15–20 mg cm^−2^ size and placed inside a controlled-environment IR transmission cell. Before pyridine adsorption, the catalyst wafer was heated to 600 °C, and the cell was degassed for 3 h. Subsequently, pyridine was introduced into the sample cell, and the cell was degassed and heated at 100 °C to remove physisorbed pyridine. The adsorbed pyridine was desorbed at 100 °C for 0.5 h, and the *in situ* FTIR spectra were obtained. The particle size of TS-1 was determined using the RISE-2008 laser particle size analyzer by dispersing 30 mg samples in 2 mL water.

### 2.4 Catalysis test

Cyclohexanone ammoximation with TS-1 catalysts was performed in a three-necked flask (100 mL) equipped with a condenser and a magnetic stirrer. TS-1 were employed as the catalyst, H_2_O_2_ aqueous solution (8 wt%, 25.8 mL) was used as the oxidant, *t*-butanol (85 wt%, 16.8 mL) was used as the solvent and aqueous ammonia (25 wt%, 13.2 mL) was used as the ammonia source. In a typical run, cyclohexanone (5.8 g), *t*-butanol and catalyst were charged in a flask. The mixture was heated to 80 °C. The reaction was then initiated by adding dilute aqueous H_2_O_2_ at a constant rate continuously using a micropump. The aqueous ammonia was added to the reaction solution in ten times. The molar ratio of precursors was as follows: *n*NH_3_H_2_O : *n*H_2_O_2_ : *n*C_4_H_10_O : *n*C_6_H_10_O = 3 : 1.05 : 2.6 : 1. After 3 h, the product mixture was analyzed by GC-7890 gas chromatography equipped with a flame ionization detector (FID) and an OV-1701 capillary column (30 m × 0.32 mm × 0.25 μm).

#### Mechanical strength test

The TS-1 powders (0.3 g) were immersed in a 2 mL 0.5% NaOH solution, which was stirred for 10 min at 80 °C. The particle size distribution was measured. The TS-1 powders were dispersed in water for comparison.

## 3. Results and discussion

To illustrate the effects of seeds on the structure and morphology of TS-1, the XRD patterns, FTIR and UV-vis spectra were obtained. The TS-1 synthesized from the inorganic reactants always had a big size.^21^

**Fig. 1:**
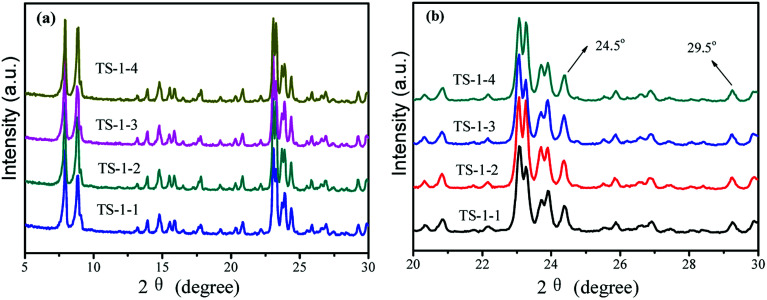
(a) XRD patterns of TS-1 and (b) enlarged version in the range from 20 to 30 degree.

**Fig. 2:**
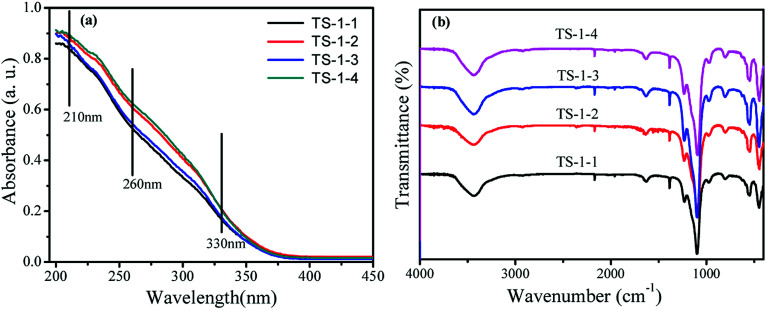
(a) UV-vis and (b) FTIR spectra of TS-1.

The crystal phase structures of TS-1 were characterized by XRD patterns (Fig. 1). There were sharp peaks at 2*θ* = 7.8°, 8.8°, 23.0°, 23.9° and 24.4°, in accordance with the five characteristic peaks of MFI topology.^19^ All TS-1 possessed a typical MFI topology structure. The single diffraction peaks at 2*θ* = 24.5° and 29.5° were ascribed to the Ti-atom entered into the zeolite framework.^42^ The RCD of TS-1-2 was highest. In the UV-vis spectra, the band around 210 nm was assigned to the framework Ti species (tetracoordinated Ti) (Fig. 2a). The bands at 260 nm and 330 nm were attributed to the amorphous Ti species (hexacoordinated Ti–O–Ti linkages and usually called non-framework Ti) and TiO_2_ anatase-like oxide phases, respectively.^43^ The band at 230 nm was attributed to the isolated Ti species with less coordination number of oxygen (such as pentahedral Ti). The UV-vis spectra indicated that all TS-1 contained tetra-coordinated Ti in the framework (Fig. 2a). For TS-1-2 and TS-1-4 with big sized seeds, the band around 260 nm suggested more non-framework Ti in the TS-1 zeolites.

All TS-1 samples possessed three typical vibration absorption peaks at 550, 800 and 960 cm^−1^ (Fig. 2b). The bands at 550 and 800 cm^−1^ were assigned to the characteristic absorption of MFI topology.^44^ The peaks at 960 cm^−1^ were attributed to the stretching vibration of [SiO_4_] units, strongly influenced by titanium ions at the neighbouring coordination sites. The TS-1 crystals had a typical MFI topology structure and framework titanium. The intensities of the bands at 960 cm^−1^ were related to the strength of the Si–O–Ti bond and the content of framework Ti. The bands at 550 cm^−1^ were attributed to the characteristic of MFI topology corresponding to the pentasil vibration of double 5-membered rings. The level of Ti atoms incorporated into the framework was estimated by the band intensity ratio *I*_960_/*I*_800_.^45^Table 1 shows that the *I*_960_/*I*_800_ ratio of TS-1-4 synthesized by the m-ZSM-5 seed is higher than those of TS-1 prepared with other seeds; this suggests more framework Ti content in the TS-1-4 with m-ZSM-5 seed.

**Table 1:** Physicochemical properties of TS-1

TS-1	RCD (%)	*I*_960_/*I*_800_	*S*_total_	*S*_mic_	*S*_ext_	*V*_total_	*V*_mic_	*V*_ext_
			m^2^ g^−1^			cm^3^ g^−1^		
TS-1-1	100	1.5	431	393	38	0.24	0.17	0.07
TS-1-2	109	1.5	458	433	25	0.24	0.19	0.05
TS-1-3	98	1.5	421	394	27	0.21	0.17	0.04
TS-1-4	101	1.6	433	402	31	0.24	0.17	0.07

**Fig. 3:**
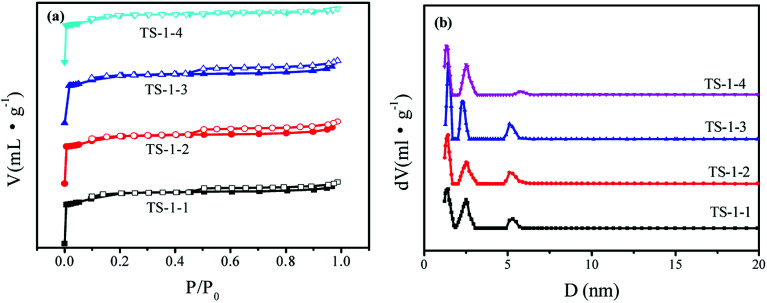
(a) N_2_-adsorption (close symbol) and desorption (open symbol) isotherms and (b) pore size distribution obtained by the DFT method of TS-1 catalysts.

N_2_-sorption isotherms were obtained to investigate the textural properties of TS-1 (Fig. 3a). There are hysteresis loops for TS-1 crystals, suggesting the mesoporous structure of TS-1 crystals; this may be due to the presence of mesopores between the crystals. The hierarchical micropores of 10 MR and mesopores of TS-1 were observed in Fig. 3b. There was a slight difference in the isotherms and pore size distribution curves. There were three main peaks in the pore size distribution, and the most probable distributions of the mesopore sizes were 5.4 nm and 2.2–2.4 nm in the four crystals. Table 1 lists the textural properties and the *I*_960_/*I*_800_ ratio of these samples. There were little differences among the *S*_BET_ of the four TS-1 samples, ranging between 421 and 458 m^2^ g^−1^. Because of the smaller crystal sizes, the *S*_ext_ of TS-1-1 with n-S-1 seed was highest among those for the TS-1 samples.

**Fig. 4:**
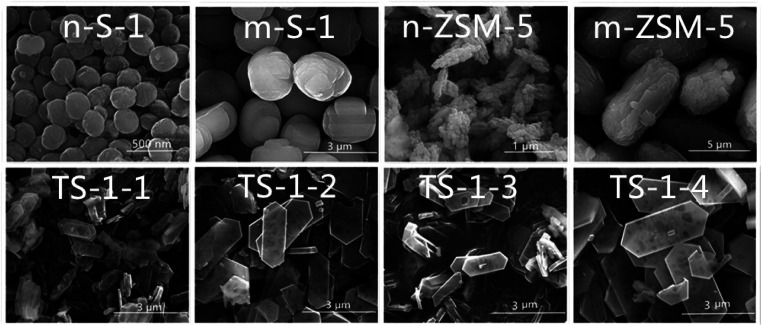
SEM images of seeds and the corresponding TS-1.

The SEM images of the TS-1 synthesized with pure silicon and aluminosilicate molecular sieve seeds are shown in Fig. 4. All TS-1 with seeds exhibited the coffin-shaped morphologies. The coffin-shaped crystals were in two regularly encountered forms.^46,47^ The crystals were enclosed by two (010) planes, two (100) planes, and four (101) planes. The pores or caves were clearly observed in the SEM images of the TS-1 crystals (Fig. 4). Twin crystals were observed to grow along the vertical direction with the (010) planes in TS-1-1 crystals, which was randomly grown together sharing the (010) plane or along the vertical direction with the (010) planes. The size and shape of the crystals were controlled by seeds. For the same type of seeds, the TS-1 sizes became larger with an increase in the seed size. The particle size of TS-1-2 was 2.32 μm when seeds with a size of 1.95 μm were added, and the size of TS-1-1 was 0.85 μm when the size of the seed was 0.25 μm. TS-1-3 and TS-1-4 with the sizes of 3.0 and 3.51 μm were obtained by adding seeds with the sizes of 0.64 and 6.59 μm, respectively.

*L*_*a*_, *L*_*b*_ and *L*_*c*_ represent the size of the MFI crystal in terms of width, depth and length, which are directly proportional to the crystal growth rates along the *a*, *b* and *c* axes, respectively (Scheme 1). The solid-phase transformation mechanism deemed the seed needed to dissolve first and functioned as a structure-directing agent in the crystallization process of TS-1 crystals. The seed was firstly dissolved to form crystal nucleus which were attached on the gel. The gel was depolymerized and reorganized into aggregates of tiny primary units, which were restructured into zeolite nuclei and then grew into irregular objects accompanying a large number of crystal intergrowths. Lastly, a bulky particle was formed as the crystallization time extended. According to the growth mechanism, the dissolution rate of seeds is the key to the nucleation, and the morphology of crystal nucleus would affect the orientation of TS-1. The dissolution of seeds became the rate-limiting step in crystal growth, and the size of the TS-1 crystals would reach minimum as the nuclei surface no longer increased at a relatively high amount of seed.^48^ The surface energies of the TS-1 planes are different, and the crystal intergrowths are facing different directions; this results in different sizes and orientations.

There were some differences between the Si/Ti ratio of the gel and that of the crystals (Table 2). The Si/Ti ratio of TS-1-2 was lower than that of other TS-1; this was ascribed to the dissolution of Si in the modification process. The Si/Ti ratios of TS-1-1 were higher because more Si from seed was introduced into the gel. The Al atom was introduced into the TS-1 crystals with the aluminosilicate zeolite seeds. The Si was dissolved with difficulty and lost from TS-1 crystals; this was attributed to the presence of Al atom in the TS-1 crystals. The Si/Ti ratios for TS-1-3 and TS-1-4 were 53.4 and 59.7, whereas the Si/Al ratios were 181.9 and 240.0, respectively. TS-1-3 synthesized with the n-ZSM-5 seed exhibited lower ratio of Si/Al and Si/Ti than TS-1-4 synthesized with m-ZSM-5 seed; this was because the Al atom in small-sized seeds more easily entered into the TS-1 framework. The hybrid atoms in the skeleton of molecular sieve were close to saturation, the more Al with less Ti was incorporated into the framework; thus, the ratio of Si/Ti in TS-1-2 was lower than that in TS-1-3 and TS-1-4.

**Table 2:** Size information and elemental analysis of the TS-1 crystals

TS-1	Seed type	Average size (μm)		Si/Ti	Si/Al	TPD (mmol g^−1^)	Molar conversion of cyclohexanone per mg Ti
		TS-1 (*L*_*c*_)	TS-1 (*L*_*b*_)				
TS-1-1	Nano S-1 (n-S-1)	0.85	0.06	69.9	—	—	0.087
TS-1-2	Micro S-1 (m-S-1)	2.32	0.18	36.0	—	—	0.127
TS-1-3	Nano ZSM-5 (n-ZSM-5)	3.00	0.23	53.4	181.9	0.11	0.146
TS-1-4	Micro ZSM-5 (m-ZSM-5)	3.51	0.34	59.7	240.0	0.06	0.117

**Fig. 5:**
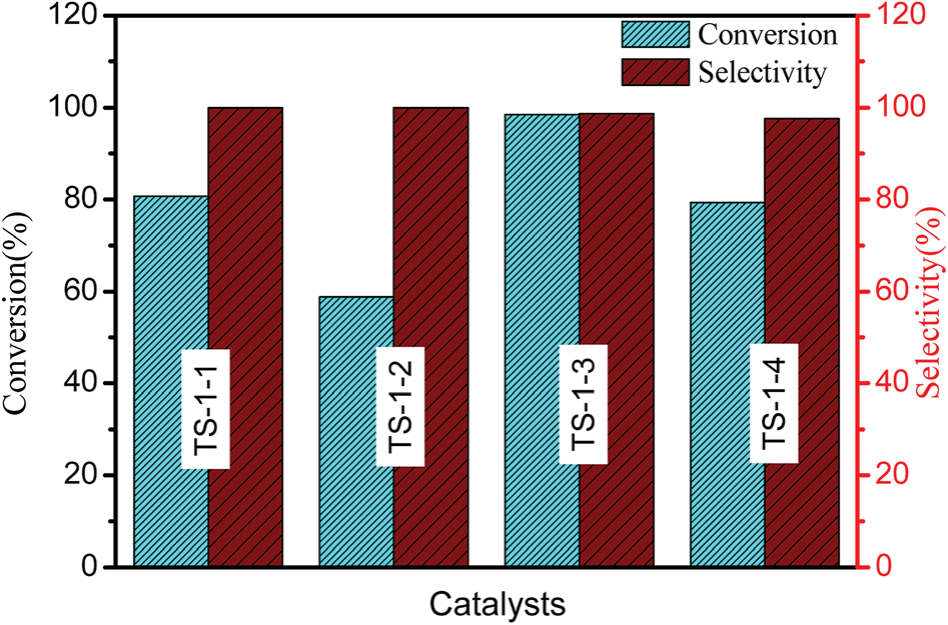
Conversion of cyclohexanone and selectivity of cyclohexanone oximes with TS-1 as catalysts in cyclohexanone ammoximation. Reaction conditions: 5.8 g of cyclohexanone with 0.40 g of catalyst at 80 °C for 3 h.

The catalytic performances of TS-1 in cyclohexanone ammoximation have been investigated (Fig. 5). TS-1-3 synthesized by nano-ZSM-5 seeds exhibited the best catalytic performances. For TS-1 prepared with the same type of seeds, higher cyclohexanone conversion was obtained over the catalysts with a smaller size, and the catalytic activity was TS-1-4 > TS-1-1 > TS-1-4 > TS-1-2, which should be attributed to the incorporation of Al atoms and thinner size of TS-1.^48^ TS-1-2, synthesized by m-S-1 seed with a size of 2.32 μm, provided worse catalytic performances than TS-1-1 in cyclohexanone ammoximation due to its high Si/Ti ratio. TS-1 synthesized by aluminosilicate seeds presented large size and high *L*_*b*_ value, but excellent catalytic activity. The possible reason was that the framework Al in the TS-1 crystals resulted in acidity, which resulted in excellent catalytic performances. The size or *L*_*b*_ value and Al in TS-1 were main reasons to cause the different catalytic performances: TS-1-3 > TS-1-1 > TS-1-4 > TS-1-2. All TS-1 synthesized with the seed presented more excellent catalytic performances than TS-1 synthesized without adding seeds (Fig. S2†).

The b-channels and a-channels are interconnected with each other, and the c-direction is a tortuous pathway. The straight channel along the *b*-axis is the fast diffusion pathway; thus, the b-oriented MFI film is mostly pursued. The molar conversion of cyclohexanone per milligram Ti (mmol mg^−1^ Ti) and the *L*_*b*_ value were calculated to explore the change tendency of cyclohexanone conversion with the variation of *L*_*b*_ ratio in Table 2. For the TS-1 with the two types of seeds, the bigger *L*_*b*_ value provided lower conversion of cyclohexanone. The molar conversion of cyclohexanone per milligram Ti of TS-1-3 with the seeds of n-ZSM-5 was higher than that of TS-1-1 and TS-1-2; this was ascribed to the implantation of Al in theTS-1 catalysts. The thinner *L*_*b*_ is conducive to the better catalytic performance. However, TS-1 catalysts with Al atoms in crystals exhibited higher *L*_*b*_ values and improved catalytic performance in cyclohexanone ammoximation due to their acidity.

**Fig. 6:**
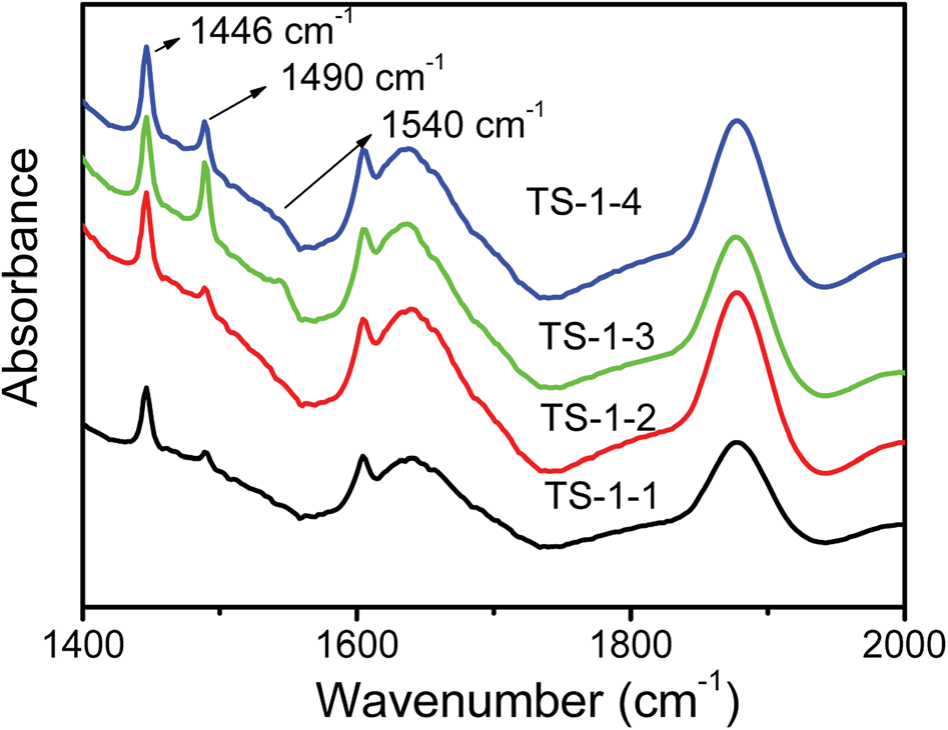
FTIR spectra of pyridine adsorption of TS-1 zeolites synthesized by aluminosilicate seeds.

The FTIR spectra of pyridine adsorbed on TS-1 zeolites after evacuation are shown in Fig. 6. The bands at 1490 cm^−1^ characteristic of both Brönsted acid sites at 1540 cm^−1^ and Lewis acid sites at 1450 cm^−1^ were assigned to Lewis acid sites;^49^ the bands at 1446 cm^−1^ associated with hydrogen-bonded pyridine were strong, and they overlapped the peak at 1450 cm^−1^ attributed to TS-1 zeolites. TS-1 zeolites synthesized by pure silicon seeds was free of Brönsted acid sites, as shown in Fig. 6; thus, the bands at 1490 cm^−1^ could infer that small amounts of Lewis acid sites existed in TS-1 zeolites synthesized by pure silicon seeds. Lewis acid and a small amount of Brönsted acid sites coexisted in TS-1 zeolites synthesized by aluminosilicate seeds, and the peak of Brönsted acid in TS-1-3 with the lowest ratio of Si/Al was the biggest among the three TS-1 zeolites, and there was a slightly weaker peak of Brönsted acid in TS-1-4 (Fig. S1†). Generally, Al atoms from seeds caused the generation of Brönsted acid.

The acidity of TS-1 zeolites synthesized by S-1 and ZSM-5 seeds was measured by NH_3_-TPD, as shown in Table 2. TS-1 synthesized with pure silica seeds exhibits a negligible NH_3_ adsorption capacity, whereas TS-1 synthesized with aluminosilicate seeds demonstrates increased NH_3_ adsorption capacity with the increasing aluminum content. These indicated that both the acid sites and the acid amounts were enhanced by introducing Al during the preparation.

**Fig. 7:**
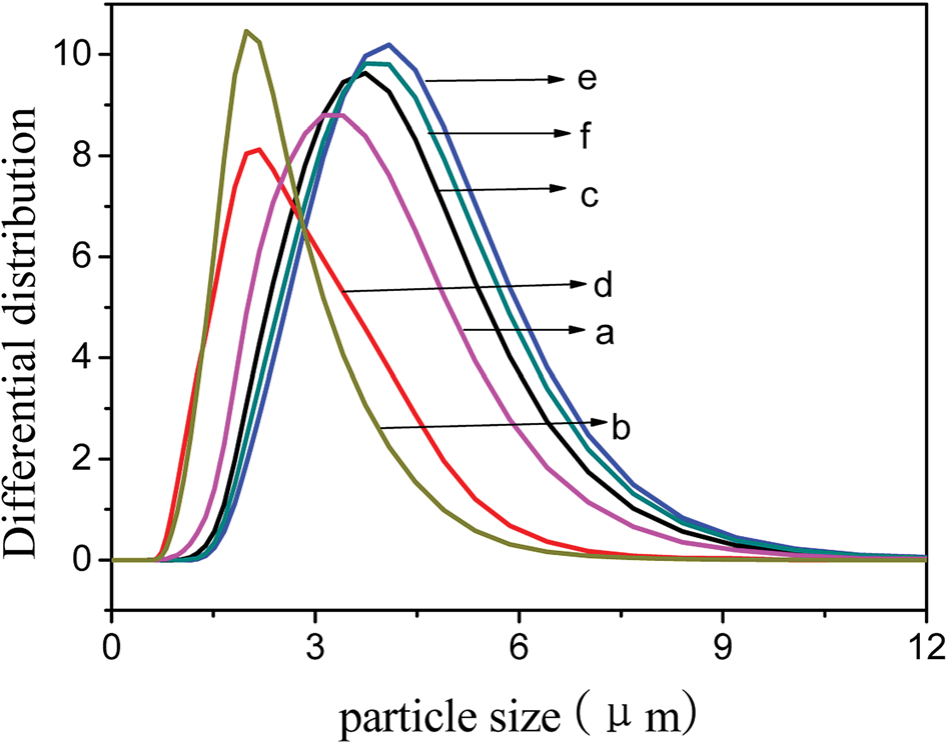
Particle size distribution of TS-1 zeolites synthesized by aluminosilicate seeds (a) TS-1-2, (b) TS-1-3, (c) TS-1-4, and destructive TS-1 (d) TS-1-2, (e) TS-1-3, and (f) TS-1-4 treated by NaOH.

The average particle size of TS-1-4 was highest and that of TS-1-1 was smallest. The particle size obtained by the SEM images was the size of *L*_*c*_, and the size obtained by the laser particle size analyzer was the size of *L*_*c*_, *L*_*b*_ or *L*_*a*_; thus, the size obtained by the SEM images was different from the size obtained by the laser particle size analyzer. The size of TS-1 decreased after treatment with NaOH solution; this implied that the crystals were corroded in a basic environment. The particle size (Fig. 7) of TS-1-4 showed little change after treatment with NaOH solution and that of TS-1-2 and TS-1-3 showed big change after the treatment. The crystals with a large *L*_*b*_ value would exhibit better mechanical strength and erosion resistance.

## 4. Conclusions

In conclusion, large-sized TS-1 crystals containing Al were synthesized with aluminosilicate zeolite as a seed in the TPAB–EA system. TS-1 crystals with different sizes and orientations were obtained by different kinds of seeds. With the decreasing size of the same type of seeds, the size of TS-1 became smaller. The depth of TS-1 zeolites affects their catalytic performances; TS-1 synthesized by seeds with Al atoms presented bigger *L*_*b*_ value and more improved catalytic performances than that synthesized with pure silica seeds. TS-1 crystals with a large *L*_*b*_ value would exhibit better mechanical strength and erosion resistance. The as-synthesized TS-1 obtained using nano ZSM-5 seed exhibited large *b*-axis depth and high catalytic performances in cyclohexanone ammoximation because the Al atom in seeds was introduced into the TS-1 crystals and brought more acid active site and strong Brönsted acid. This study is of great significance for the design of high-performance industrial catalysts.

## Conflicts of interest

There are no conflicts to declare.

## Supplementary Material

RA-009-C8RA10104C-s001
