# Supplementary material for: Seed-assisted synthesis of TS-1 crystals containing Al with high catalytic performances in cyclohexanone ammoximation
Source: RSC Adv. 2019 Jan 18;9(5):2386–94. doi: 10.1039/c8ra10104c (PMC9059887; doi:10.1039/c8ra10104c)
Supplement: RA-009-C8RA10104C-s001 [file RA-009-C8RA10104C-s001.pdf]

Electronic Supplementary Material (ESI) for RSC Advances.
This journal is © The Royal Society of Chemistry 2018

Supporting Information for
Seeds-assisted synthesis of TS-1 crystals containing Al with high catalytic performances in cyclohexanone ammoximation

Yan Xue, *[a] Guangling Zuo,[a] Yiqiang Wen, [b] Huijuan Wei, [b] Meng Liu, [b] Xiangyu Wang*[b] and Baojun Li[b]

[a] *School of Biological and chemical engineering, Nanyang Institute of Technology, 80 Changjiang Road, Nanyang 473004, China.*

b Institute of Industrial Catalysis, School of Chemistry and Molecular Engineering, Zhengzhou University, 100 Science Road, Zhengzhou 450001, China.

* Correspondence Author. Tel.: +86-15688121688, +86-13526785218.

E-Mail address: yanxue800@163.com, wangxiangyu@zzu.edu.cn

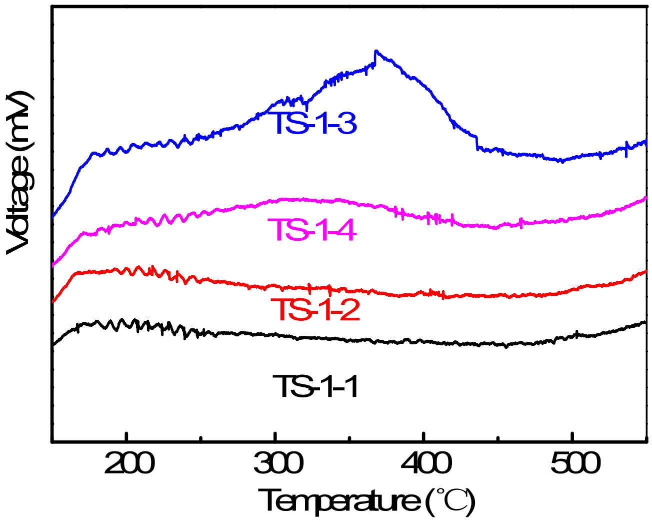

Figure S1. NH_3-TPD of TS-1 syntheszed with differet seed.

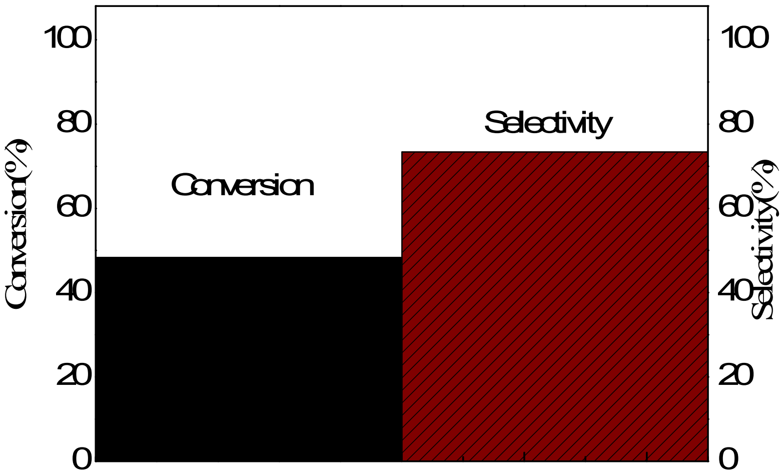

Figure S2. The cyclohexanone conversion and cyclohexanone oxime selectivity of TS-1 without adding seed. Reaction conditions: 5.8 g of cyclohexanone with 0.40 g of catalyst at 80 °C for 3 h.
